# Risk factors, prevention, and therapy of intraluminal stent thrombosis in frozen elephant trunk prostheses—what we know so far

**DOI:** 10.3389/fcvm.2024.1344292

**Published:** 2024-03-13

**Authors:** Florian Helms, Bastian Schmack, Alexander Weymann, Reza Poyanmehr, Andreas Martens, Jawad Salman, Alina Zubarevich, Jan D. Schmitto, Arjang Ruhparwar, Aron-Frederik Popov

**Affiliations:** Division for Cardiothoracic-, Transplantation- and Vascular Surgery, Hannover Medical School, Hannover, Germany

**Keywords:** intraluminal stent thrombosis, stent graft thrombosis, frozen elephant trunk (FET), aortic surgery, aortic arch replacement

## Abstract

Intraluminal thrombus formation (ILT) is a recently discovered and highly clinically relevant complication after frozen elephant trunk implantation in cardiovascular surgery. In this phenomenon, a thrombus forms within the lumen of the stent graft component of the frozen elephant trunk prosthesis and puts the patient at risk for downstream embolization with visceral or lower limb ischemia. Incidence of ILT reported in the currently available studies ranges from 6% to 17% of patients after frozen elephant trunk implantation. Adverse thromboembolic events include acute occlusion of the celiac and superior mesenteric arteries, both renal arteries as well as acute lower limb ischemia due to iliac or femoral artery embolization that not infrequently require interventional or open embolectomy. Therefore, the presence of ILT is associated with increased short-term mortality and morbidity. Currently proposed strategies to avoid ILT formation include a more aggressive anticoagulation management, minimization of postoperative coagulation factor application, and even technical optimizations of the stent graft portion itself. If ILT is manifested, the therapeutic strategies tested to date are long-term escalation of anticoagulation and early endovascular extension of the FET stent graft with overstenting of the intraluminal thrombus. The long-term efficiency of these prophylactic and therapeutic measures has yet to be proven. Nonetheless, all surgeons performing the frozen elephant trunk procedure must be aware of the risk of ILT formation to facilitate a timely diagnosis and therapy.

## Introduction

Around the turn of the millennium, the elephant trunk prosthesis originally developed by Borst et al*.* in 1983 (1) was refined into the frozen elephant trunk (FET) by implementing a stented endovascular prosthetic component (2, 3). Today, the frozen elephant trunk procedure has become the standard approach for extensive aortic arch and proximal descending aorta replacement (4, 5). For this, the most commonly used commercially available devices are the JOTEC- E-vita (JOTEC GmbH, Hechingen, Germany) and the VASCUTEC Thoraflex™ (VASCUTEK, Terumo, Inchinnan, Scotland, United Kingdom) (6). Compared to the classical elephant trunk technique, the endovascular stent graft of the frozen elephant trunk facilitates occlusion of entry tears located in the proximal descending aorta in type A- as well as in complicated type B- dissections (7–9), prevents aneurysm expansion in the downstream aorta (10–12), and provides an excellent landing zone for secondary endovascular stent elongations (13). With these features, the establishment of the frozen elephant trunk prosthesis undoubtedly marks a significant milestone for the treatment of complex pathologies of the aortic arch. However, with increasing use, complications of this comparatively young technique are now becoming apparent. An only recently discovered problem is intraluminal thrombus (ILT) formation in the endovascular stent graft component of frozen elephant trunk prostheses. This narrative review gives an overview of the incidence, diagnostics, and risk factors of intraluminal thrombus formation as well as proposed clinical measures to avoid it.

## ILT in endovascular aortic stent grafts

Although it is just now becoming apparent in FET prostheses, ILT formation in stented aortic grafts *per se* is not a new phenomenon and has been described for endovascular thoracic and abdominal aortic grafts even before the frozen elephant trunk technique was widely used (14–19). Here, an incidence of ILT ranging from 10% to 36% is reported (16, 17). ILT in endografts was frequently detected as a late complication during long-term follow-up with new thrombus formation occurring even after more than 24 months after implantation (16). In a multivariate logistic regression analysis, Russell et al*.* identified preexisting peripheral artery disease as a risk factor for ILT formation after endovascular aortic aneurysm repair (17). In their experience, escalation of antithrombotic therapy was associated with regression or prevention of progression of ILT in endovascular aortic grafts.

## Definition and diagnostics of ILT

Here, corresponding to the definitions used for thrombus formation in endovascular stent grafts, ILT was defined as an endograft thrombus formation with a minimum thickness of 2 mm and a minimum longitudinal length of 4 mm (16). Diagnosis is usually made by contrast enhanced computed tomography, which is recommended for long-term follow up in current guidelines (20). As usual in the analysis of aortic pathologies, all measurements should be made in multiplanar reconstruction and strictly perpendicular to the aortic axis (21). Although not explicitly stated in practice guidelines, many centers perform standard pre-discharge CT-angiography especially with a focus on early ILT formation (21–23). If CT-angiography is inconclusive, Martens et al*.* propose the use of transesophageal echocardiography of the descending aorta (22). While visualization of the aortic arch is limited, transesophageal sonography allows excellent analysis of the descending stent graft and provides evaluation of thrombus mobility throughout the cardiac cycle by live video imaging. In earlier studies, transesophageal echocardiography has been shown to be a sensitive diagnostic tool for the evaluation of the descending aorta and detection of aortic atheroma (24, 25).

## Incidence and characteristics of postoperative ILT

In the currently available studies focusing specifically on ILT formation, reported incidence of postoperative ILT after the frozen elephant trunk procedure ranges from 6% to 16.8% of cases (21, 23) (Table 1). Contrary to that, this phenomenon has not been reported in the initial pioneering experience reports of the frozen elephant trunk technique (26, 27). Thus, it is very likely that this complication that is just now moving into the center of attention has been underdiagnosed in the first series. In the currently available reports, diagnosis of ILT is usually made in the postoperative pre-discharge CT-angiography within the first seven days after FET-implantation. Contrary to that, late occurrence of a new ILT in the long-term follow up CT-scans was not described in the currently available reports. Thus, ILT formation seems to occur within the first postoperative days.

**Table 1:** Best evidence studies.

** **	Walter et al*.* (21)	Martens et al*.* (22)	Misfeld et al*.* (23)
Number of patients	304	281	125
Incidence of ILT	6%	8.2%	16.8%
Time from operation to ILT diagnosis	7 days	4.6 days	N/A
Postoperative anticoagulation protocol	Heparinization for 6 h postoperatively, subsequently anti-platelet therapy (ASS)	Anti-platelet therapy (ASS) + low-dose heparin (thrombosis prophylaxis)	Heparinization for 6 h postoperatively, subsequently anti-platelet therapy (ASS) + low-dose heparin
ILT location	• 21% proximal half of the stent • 79% distal half of the stent	• Inner curvature • Distal stent end	N/A
Risk factors	• Age^a^ • Female sex^a^ • Aortic aneurysm^a^	• Increased stent graft diameter index^a^ • Degenerative aneurysm^a^ • Larger aortic diameter • Anticipated Type Ib Endoleak • HIT	• Incomplete aneurysmal stent coverage • FET stent diameter <34mm • Higher volumetric size of the descending aorta • → smaller stent-to-aneurysm diameter ratio • Conservative management of major postoperative bleeding
Clinical complications	• 21% of patients with ILT developed embolic complications	• ILT Formation as an independent risk factor for perioperative mortality • 27% of patients with ILT developed embolic complications requiring re-intervention or operation	• ILT was associated with higher in-hospital mortality*
Therapeutic management	• Therapeutic anticoagulation • 20% TEVAR	• 18% no treatment • 55% therapeutic anticoagulation (VKI or DOAK) • 27% interventional/ surgical embolectomy	• Therapeutic anticoagulation (VKI)
Recommendations	• Perform postoperative surveillance by CTA in all patients following FET implantation	• Consider therapeutic anticoagulation in patients at risk of ILT. • Perform early postoperative CTA or TEE to rule out ILT (within 10 days). • In patients developing ILT consider early TEVAR extension.	• Consider early postoperative intravenous anticoagulation in all FET-patients • Consider extended oral anticoagulation in patients with high thrombosis risk • Consider early surgical re-intervention for bleeding • Avoid prolonged conservative bleeding management and coagulation factor administration

^a^ Independent risk factors in multivariate regression analysis.

* 19% vs. 8.7% *p* = 0.3.

**Figure 1:**
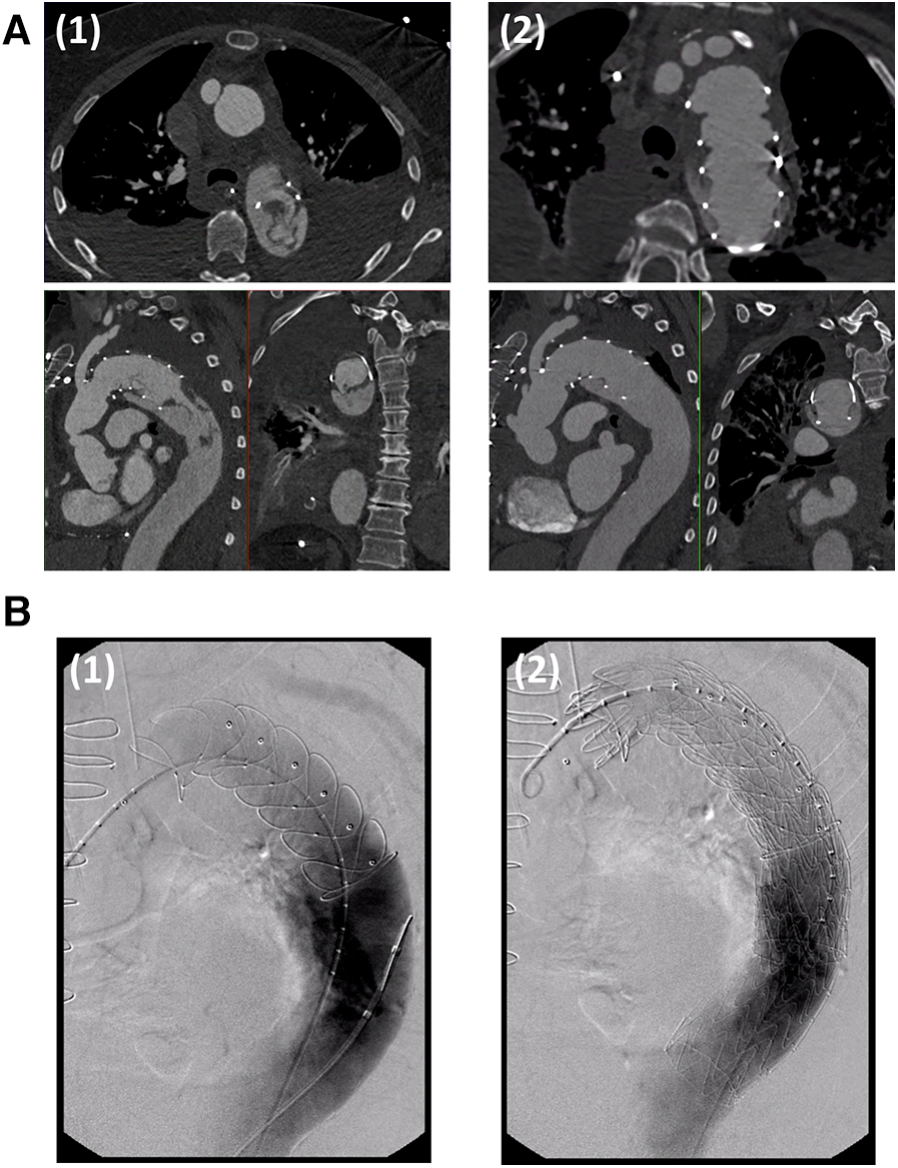
(**A**) CT-angiography of a patient with an intraluminal thrombus located at the distal end of the frozen elephant trunk stent graft diagnosed on the third postoperative day (1). Oral anticoagulation with a vitamin K inhibitor resulted in complete thrombus remission within 40 days (2). (**B**) TEVAR extension in a patient with mega-aortic syndrome and intraluminal thrombus formation after frozen elephant trunk implantation. The frozen elephant trunk stent graft is probed via a transfemoral access. Angiography showed an expected Type 1b endoleak (1). After TEVAR-extension, the endoleak was sealed and the intraluminal thrombus was excluded completely (2). Figures were adapted with permission from Martens et al.

Walter et al*.* reported that thrombi were predominantly located in the distal half of the FET stent portion (21). Likewise, Martens et al*.* identified the distal end of the graft as a typical location of ILT ranging into the native descending aorta (22) (Figure 1A). Moreover, they observed that the thrombi were predominantly located at the inner curvature of the stent graft and propose that stent graft pockets resulting from graft folding around the ring stents in the inner curvature of the distal aortic arch may be a predisposed location for ILT formation due to low- and no-flow areas within the prosthesis. This theory is supported by previously published experiences concerning ILT formation in thoracic endovascular aortic repair (TEVAR), which is also proposed to be attributable to endograft infolding (28, 29).

## Risk factors

Concerning the indication for FET-implantation, there is a broad consensus among the currently published studies that thoracic aneurysms are associated with a greater risk for ILT compared to patients with aortic dissections (21–23). Here, Walter et al*.* identified descending aortic aneurysms as an independent risk factor in multivariate regression analysis (21). Furthermore, the Freiburg group around Misfeld et al*.* observed a higher volumetric size of the descending aorta in patients who developed ILT (23). Both, Martens et al*.* and Misfeld et al*.* reported higher incidences of incomplete aneurysmal stent coverage and expected type I b endoleak in patients with ILT-formation (22, 23). The latter group also found that a lower stent-to-aneurysm diameter ratio was associated with FET endograft thrombosis. Thus, they propose turbulent flow and stasis at the intersection of a small stent graft and a large aneurysm sac as a potential mechanism of ILT formation at the distal end of the stent portion. Contrary to these findings, Martens et al*.* reported that an increased stent graft diameter in relation to the patients body height was associated with ILT formation. However, they too consider stasis to be a major mechanistic factor for development of endograft thrombosis. In this case, stasis is proposed to result from slower flow velocity within the large graft compared to body size.

Concerning perioperative coagulation management, Misfeld et al*.* observed a higher incidence of ILT in patients in which major postoperative bleeding was managed conservatively. They conclude that liberal administration of coagulation factors and hemoderivates may promote ILT formation. Contrary to that, intraoperative administration of coagulation factors, platelet concentrates, and fresh frozen plasma was not associated with increased rates of ILT in the Hannover experience published by Martens et al*.* (22). The standard postoperative coagulation management was comparable in all three studies. While Walter et al*.* and Misfeld et al*.* performed heparinization for the first 6 h postoperatively before starting anti-platelet therapy with ascorbic acid, Martens et al*.* used low-dose heparin and anti-platelet therapy alone. Despite omission of early postoperative heparinization, ILT incidence was not distinctively higher compared to the other two studies.

As an additional risk factor, Martens et al*.* observed a higher rate of ILT in patients that developed heparin induced thrombocytopenia. Another example for the association of a hypercoagulable state with ILT formation is provided by Walter et al*.* who reported thrombus formation in a patient with Factor-V-Leiden mutation. Additionally, they reported female sex and higher age as independent risk factors for ILT formation (21). Again, the former was also previously identified as a risk factor for distal narrowing of TEVAR-grafts (30).

As a clinical tool to identify patients at higher risk for ILT formation after FET implantation, Martens et al*.* proposed a score based on the Virchow's triad of risk factors for peripheral thrombus formation, which is therefore referred to as the “Virchow score” (22). In this scoring system, the stent graft diameter relative to the patient's body height (+1 point if >20 mm/m) and the presence of HIT (+2 points) as well as therapeutic anticoagulation as a protective factor (−1 point) were considered. Although it has not been formally validated yet, the “Virchow score” was significantly higher in patients that developed ILT compared to patients without ILT in the experience of Martens et al*.* (22).

## Impact of the graft type on ILT formation

While Martens et al*.* and Walter et al*.* worked exclusively with the VASCUTEC Thoraflex™ prosthesis, Misfeld et al*.* used both, the Thoraflex and the JOTEC- E-vita and provided a subgroup analysis regarding the potential impact of the device type on ILT formation. Here, no significant differences were found with respect to the incidence of ILT or other adverse postoperative events. However, taking together all three available studies, it should be noted that the overall number of patients treated with the JOTEC graft is comparatively low. Thus, currently reported studies are not powered to reliably exclude potential differences between the two devices with respect to ILT formation. Nonetheless, there are distinct differences between the two devices regarding the design of the stent part of the prostheses: While curved nitinol rings are used in the Thoraflex™ prostheses, the JOTEC- E-vita graft is supported by classical z-shaped nitinol stents as they are used frequently in established TEVAR and EVAR grafts as well. These different designs may affect the formation of graft pockets with slow-flow zones, which have been proposed to be associated with ILT formation (22).

## Clinical consequences of ILT

The clinical relevance with respect to potential thromboembolic or other adverse events associated with ILT is still not well understood. Complementary evidence can be derived again from the reports of the already longer known ILT formations in endovascular aortic stents. Here, Maleux et al*.* report ILT formation occurring in up to one third of patients undergoing endovascular abdominal aortic repair (EVAR) but no clinically apparent distal thromboembolic complications in midterm follow-up (15). These results were later confirmed by Massoni et al*.* in 2020, who as well found no significant correlation between ILT and thromboembolic events in 221 patients after EVAR (16). Contrary to that, Russell et al*.* reported that 23% of patients with ILT after EVAR presented with symptoms of lower body claudication and 8% even required re-intervention for acute limb ischemia (17).

While the embolization rates in EVAR patients with ILT are comparatively low and reinterventions for ILT- associated complications are very rarely necessary, endoluminal thrombi in FET prosthesis appear to cause clinically relevant complications more frequently: In the experience of Martens et al*.*, ILT was identified as an independent risk factor for perioperative mortality. In consonance with these findings, Misfeld et al*.* reported an in-hospital mortality of 19% in patients with ILT compared to 8.7% in patients without ILT, although this did not formally reach statistical significance (*p* = 0.3) and the study was not designed to proof direct causal connection between ILT and mortality. Nonetheless, thromboembolic events may have contributed to specific organ failure in these cases. Furthermore, embolic complications associated with ILT seem to be by far more common in patients undergoing FET-Implantation compared to the reported embolization rates of EVAR-patients. Here, thromboembolic events are reported in 21% of patients with ILT in the experience of Walter et al*.*, while the Hannover group reported thromboembolic adverse events in 27% of patients who developed endoluminal thrombosis after FET implantation. Embolic total and subtotal occlusions were reported for the celiac trunk, the superior mesenteric artery, and both renal arteries (21) as well as for the lower limbs (22).

## Prophylactic measures to prevent ILT

Following the risk factor analysis and considering the experience in the clinical management of ILT in FET- prostheses, some recommendations for the prevention of ILT can already be deducted from the currently available pioneering studies on this very novel complication: First, extended oral anticoagulation for up to 3 months postoperatively until the next follow up CT-scan is recommended by Martens et al*.* as well as by Misfeld et al*.* (22, 23). Supporting this strategy, perioperative anticoagulation initiated for other indications (e.g., atrial fibrillation) has been shown to be a protective factor against ILT formation (22). With the same rationale, Misfeld et al*.* strongly advise against prolonged conservative bleeding management with extensie clotting factor administration. Instead, they emphasize the paramount importance of meticulous intraoperative hemostasis and recommend a more liberal indication for surgical re-exploration in case of extensive postoperative bleeding (23). If the risk for embolization is high or if the thrombus persists despite extended oral anticoagulation, TEVAR extension of the FET-prosthesis with over-stenting of the endograft thrombus may also be considered (21–23) (Figure 1B). In the currently available reports, over-stenting of the intraluminal thrombus with endovascular stents facilitated reliable thrombus exclusion.

In addition to these clinical measures, the authors of the best-evidence studies also see potential for technical improvements of the FET prosthesis itself: With reference to *in vitro* particle image velocimetry investigations indicating unphysiological flow and stasis at the distal end of the FET stent grafts in descending aortic aneurysms (31), Misfeld et al*.* call for a biomechanical optimization of the FET prosthesis design with regard to physiological flow conditions, especially at the distal end of the stent portion (23). Furthermore, Martens et al*.* developed a custom-designed modification of the Thoraflex™ FET-prosthesis with interposed additional ring stents that are angled relative to the original stents (22). The rationale behind this new design is to reduce the size of the stent graft pockets resulting from banding of the stent graft and thus minimize slow- and no-flow zones especially in the inner curvature of the graft. Similarly, the z-stent design used in the E-vita graft and endovascular grafts may reduce the graft pocket sizes and with that prevent slow flow areas.

## Clinical management of ILT

There is a broad consensus among all three best-evidence authors that a pre-discharge CT-angiography with a special focus on potential ILT should be performed in all patients after the FET procedure. The first and most commonly recommended clinical measure to address manifested ILT in patients after frozen elephant trunk implantation was initiation of therapeutic anticoagulation. Initially, therapeutic heparinization and subsequently, oral anticoagulation using vitamin-K-inhibitors (22, 23) or direct acting oral anticoagulants (22) were recommended. In symptomatic patients or when embolic complications were diagnosed, open or interventional embolectomy and endovascular stent graft extension by TEVAR were performed. While Walter et al*.* reported successful thrombus remission without adverse embolic events under oral anticoagulation alone (21), Martens et al*.* observed embolic complications in 20% of patients treated solely with therapeutic anticoagulation. Likewise, Walter et al*.* report one case in which therapeutic anticoagulation did not result in thrombus regression. This patient was then successfully treated using a conventional aortic stent-graft. There are currently no reports on medical thrombolytic therapy for ILT in the available literature to date. While this strategy has been reported to be successful for native aortic thrombosis (32), patients with ILT have a considerably higher bleeding risk or even contraindication for thrombolytic therapy especially in the first postoperative days. Thus, the risk of thrombosis, ischemia and bleeding must be carefully evaluated and weighted when determining the indication for anticoagulation or even lysis therapy.

## Discussion

Intraluminal stent thrombosis is a highly relevant complication associated with the frozen elephant trunk procedure that may has been underdiagnosed and underestimated in the first years of the broad clinical application of the frozen elephant trunk for total aortic arch replacement. Unlike what was observed in classical endovascular aortic stents, ILT formation in the FET prosthesis is often complicated by embolic events and has a negative impact on short-term survival and morbidiy. Although ILT is an only very recently discovered complication of the frozen elephant trunk procedure, risk factors associated with ILT formation can all be attributed to the long-known Virchow's triad for peripheral thrombus formation: disruption of the intimal lining, stasis, and presence of a hypercoagulable state (33). Since the commercially available FET prostheses consist of the synthetic matrix material polyethylene terephthalate (Dacron®) with limited hemo- and biocompatibility (34) and have large areas of intravascular exposed foreign surfaces, the first Virchow's criterion of a disrupted endothelial lining applies by default. Furthermore, stasis as the second criterion of the Virchow's triad has been identified as a potent risk factor for ILT formation. Here, stasis can result from pockets developing due to folding of the stent graft itself (22) or may be caused by size mismatch and slow- and no-flow areas at the distal end of the FET stent especially in larger descending aortic aneurysms with type 1B endoleaks (23). Concerning the hypercoagulable state, the presence of heparin induced thrombocytopenia as well as the Factor-V-Leiden mutation have been shown to be associated with ILT formation (21, 22). Thus, they complete the three detectable criteria of the Virchow's triad in the FET associated ILT formation. Taking these observations into account, the “Virchow-score” proposed by Martens et al*.* may be a useful tool to detect patients that are at a particularly high risk for ILT formation, although this scoring system has yet to be clinically validated (22). While comparative studies between the Thoraflex and the E-vita graft are overall scarce, no relevant differences between the two grafts have become apparent to date (23).

With regard to practical clinical measures, building on the evidence available to date, it can be stated that every patient undergoing FET implantation should receive a pre-discharge CT-angiography to detect early endograft thrombus formation. Since ILT seems to develop within the first postoperative days, routine CT-angiography at the end of the first postoperative week appears reasonable. If ILT is detected, authors of the best evidence studies uniformly recommend escalation of the anticoagulation strategy with subscription of oral anticoagulants post discharge and close follow-up CT-angiography. In some of the reported cases, oral anticoagulation alone resulted in complete remission even of complex thrombi (Figure 1A). If this strategy fails or an extremely high risk of embolization is predicted, early endovascular extension of the FET stent may also be considered, which appears to safely exclude the intraluminal thrombus (Figure 1B).

Since ILT is a comparatively newly discovered complication, there is still insufficient evidence, particularly with regard to the optimal therapeutic strategy, at the current stage. Consequently, no official adverse effect report from the regulatory institutions regarding ILT in FET-prostheses have been released to date. Future studies are required to determine the optimal standard perioperative anticoagulation strategies for all patients undergoing FET Implantation and evaluate which long-term therapeutic strategy is the safest and the most efficient if manifested ILT is diagnosed.

First and foremost, surgeons and centers performing the frozen elephant trunk procedure must be aware of ILT as a new and highly relevant complication associated with increased mortality and morbidity in patients with FET prostheses in order to facilitate a timely diagnosis and initiate appropriate clinical measures.
